# Adverse Pregnancy Outcomes in Solid Organ Transplant Recipients: A Systematic Review and Meta-Analysis

**DOI:** 10.1001/jamanetworkopen.2024.30913

**Published:** 2024-08-29

**Authors:** Jennifer H. Yo, Neville Fields, Wentao Li, Alice Anderson, Sarah A. Marshall, Peter G. Kerr, Kirsten R. Palmer

**Affiliations:** 1 Department of Nephrology, Monash Health, Melbourne, Victoria, Australia; 2 Monash Women’s and Newborn, Monash Health, Melbourne, Victoria, Australia; 3 Department of Obstetrics & Gynaecology, Monash University, Melbourne, Victoria, Australia; 4 National Perinatal Epidemiology and Statistics Unit, Centre for Big Data Research in Health and School of Clinical Medicine, Faculty of Medicine, University of New South Wales, Sydney, New South Wales, Australia; 5 Library Services, Monash Health, Melbourne, Victoria, Australia; 6 Department of Medicine, School of Clinical Sciences, Monash University, Melbourne, Victoria, Australia; 7 The Ritchie Centre & the Hudson Institute of Medical Research, Department of Obstetrics and Gynaecology, Monash University, Melbourne, Victoria, Australia

## Abstract

**Importance:**

Transplant recipients experience high rates of adverse pregnancy outcomes; however, contemporary estimates of the association between solid organ transplantation and adverse pregnancy outcomes are lacking.

**Objective:**

To evaluate the association between solid organ transplantation and adverse pregnancy outcomes and to quantify the incidence of allograft rejection and allograft loss during pregnancy.

**Data Sources:**

PubMed/MEDLINE, EMBASE and Scopus databases were searched from January 1, 2000, to June 20, 2024, and reference lists were manually reviewed.

**Study Selection:**

Cohort and case-control studies that reported at least 1 adverse pregnancy outcome in pregnant women with solid organ transplantation vs without solid organ transplant or studies that reported allograft outcomes in pregnant women with solid organ transplantation were included following independent dual review of abstracts and full-text articles.

**Data Extraction and Synthesis:**

Two investigators abstracted data and independently appraised risk of bias using the Newcastle Ottawa Scale. A random-effects model was used to calculate overall pooled estimates using the DerSimonian-Laird estimator. Reporting followed the Meta-analysis of Observational Studies in Epidemiology (MOOSE) reporting guideline.

**Main Outcomes and Measures:**

Primary pregnancy outcomes were preeclampsia, preterm birth (<37 weeks), and low birth weight (<2500 g). Secondary pregnancy outcomes were live birth rate, gestation, very preterm birth (<32 weeks), very low birth weight (<1500 g), and cesarean delivery. Allograft outcomes were allograft loss and rejection during pregnancy.

**Results:**

Data from 22 studies and 93 565 343 pregnancies (4786 pregnancies in solid organ transplant recipients) were included; 14 studies reported adverse pregnancy outcomes, and 13 studies provided data for allograft outcomes. Pregnancies in organ transplant recipients were associated with significantly increased risk of preeclampsia (adjusted odds ratio [aOR], 5.83 [95% CI, 3.45-9.87]; *I*^2^ = 77.4%), preterm birth (aOR, 6.65 [95% CI, 4.09-12.83]; *I*^2^ = 81.8%), and low birth weight (aOR, 6.51 [95% CI, 2.85-14.88]; *I*^2^ = 90.6%). The incidence of acute allograft rejection was 2.39% (95% CI, 1.20%-3.96%; *I*^2^ = 68.5%), and the incidence of allograft loss during pregnancy was 1.55% (95% CI, 0.05%-4.44%; *I*^2^ = 69.2%).

**Conclusions and Relevance:**

In this systematic review and meta-analysis, pregnancies in recipients of a solid organ transplant were associated with a 4 to 6 times increased risk of preeclampsia, preterm birth, and low birth weight during pregnancy. There was a low overall risk of graft rejection or loss during pregnancy.

## Key Points

**Question:**

Is solid organ transplantation associated with an increased risk of adverse pregnancy outcomes?

**Findings:**

In this systematic review and meta-analysis of 22 studies with 93 565 343 pregnancies, there was a statistically significant 4- to 6-fold increased risk of preeclampsia, preterm birth at less than 37 weeks, and low birth weight of less than 2500 g in pregnancies with a solid organ transplant compared with pregnancies without a solid organ transplant.

**Meaning:**

These findings suggest that pregnancies in solid organ transplant recipients have increased risk of adverse outcomes.

## Teaser

This systematic review and meta-analysis evaluates the association of solid organ transplantation with adverse pregnancy outcomes compared with pregnancy in individuals without solid organ transplantation and assesses the incidence of allograft rejection or loss during pregnancy.

## Introduction

During the 21st century, the rate of solid organ transplantation has increased dramatically, resulting in an increasing number of women of childbearing age with kidney, liver, heart, or lung transplants.^1,2,3^ In the US, there were 65 775 organ transplants in 2023, of which 9077 were kidney transplants and 3116 were liver transplants in women aged 18 to 49 years.^1^ Among transplant recipients, pregnancy is associated with a disproportionately increased risk of adverse pregnancy outcomes, including preeclampsia, low birth weight, and premature birth.^4,5,6^

Much of our understanding of the risks during pregnancy are derived from registry and single-center reports. Previous meta analyses have reported pooled incidences of various pregnancy outcomes in both kidney and liver transplant recipients.^4,5,6^ However, to our knowledge, none have measured the strength of association between adverse pregnancy outcomes and solid-organ transplantation compared with pregnancies without transplantation. The estimation of the risk of developing adverse pregnancy outcomes provides valuable data with which to inform prepregnancy counselling and enhance interdisciplinary antenatal care.

As such, the primary goal of this study was to synthesize the available evidence on the associations between solid organ transplantation and adverse pregnancy outcomes. Our secondary goal was to examine the impact of pregnancy on the allograft by summarizing the incidence of acute allograft rejection and loss during pregnancy.

## Methods

For this systematic review and meta-analysis, a prespecified protocol was registered with PROPSERO (CRD42021269591). Institutional review board approval was not required for this meta-analysis because it included only previously published research. Reporting for this study adheres to the Meta-analysis of Observational Studies in Epidemiology (MOOSE) reporting guideline.^7^

### Data Sources and Search Strategy

A systematic search of PubMed/Medline, EMBASE and Scopus was undertaken from January 1, 2000, to June 20, 2024, by a research librarian (A.A.). The search was limited to articles from 2000 onward to represent more current populations. This was supplemented by hand-searching reference lists of key citations. For the primary goal, we sought studies with the following: a population of pregnant individuals with solid organ transplantation (kidney, liver, heart, or lung), a comparison group of pregnant individuals with no solid organ transplantation, and at least 1 primary or secondary pregnancy outcome of interest. For the secondary goal, we also included studies that reported allograft loss or allograft rejection during pregnancy in pregnant individuals with a solid organ transplant. A detailed search strategy is available in eAppendix 1 in Supplement 1. While the search and eligibility criteria used an inclusive definition of women (ie, pregnant populations that included all gender identities), all studies that we found referred to their populations as women.

### Outcomes

The primary pregnancy outcomes were preeclampsia, preterm birth (ie, <37 weeks’ gestation), and low birth weight (ie, <2500 g). Secondary pregnancy outcomes were gestational age at birth in weeks, live birth, very preterm birth (ie, <32 weeks’ gestation), very low birth weight (ie, <1500 g), and cesarean delivery. For the secondary goal, the allograft outcomes were acute allograft rejection during pregnancy and acute allograft loss during pregnancy.

### Study Selection

Peer-reviewed, English-language, cohort and case-control studies were included if they published original effect estimates of the association between solid organ transplantation and at least 1 primary or secondary pregnancy outcome or reported the risk of allograft outcomes during pregnancy in solid organ transplant recipients. Independent dual review of abstracts and full text articles was performed by 3 authors (J.H.Y., N.F., or S.A.M.). Discrepancies in study selection were resolved by 2 mediators (K.R.P. and P.G.K.). If the same registry or patient population was used in more than 1 study, the larger of the studies was included.

### Data Extraction and Risk of Bias Assessment

Two reviewers (J.H.Y., N.F., or S.A.M.) independently extracted data from eligible studies. Where information was not available from the studies, authors were contacted to request this information. Risk of bias was independently dual rated using the Newcastle-Ottawa Scale (NOS) for observational studies.^8^ Based on the NOS, studies were classified as high (8-9 stars), moderate (6-7 stars), and low (<6 stars) risk of bias. For studies that reported allograft outcomes only, a modified NOS using selection and outcome was used as the category for comparability was deemed irrelevant for this outcome. For the modified NOS, studies were judged to be low risk of bias (≥4 stars) and high risk of bias (<4 stars). Discrepancies in data extraction and quality appraisal were resolved through consensus with a third reviewer.

### Statistical Analysis

Random effects meta-analysis using the DerSimonian-Laird method was used to calculate overall pooled odds ratios (ORs) of the association between solid organ transplantation and primary or secondary adverse pregnancy outcomes.^9^ Adjusted ORs (aORs) from studies that adjusted for at least 1 confounding factor were included in the meta-analysis. If aORs were unavailable, crude estimates were pooled separately. Incidence rate ratio and risk ratio were used as approximations of each other in the setting of low incidence rate^10^ and the aOR was computed.^11^ For continuous outcomes, the mean difference (MD) was calculated. Pooled incidences were expressed as proportions after transforming data using Freeman-Tukey Double Arcsine transformation.^12^ Heterogeneity was assessed using τ^2^ statistic and *I*^2^ values.

Subgroup analysis by solid organ transplant type and maternal factors were planned but not conducted owing to the small number of included studies. Tests of small study effects were evaluated using the Egger test^13^ and funnel plots for outcomes with at least 10 studies.^14^

Sensitivity analyses with restriction of the meta-analyses to studies reporting effect estimates adjusted for important confounding factors specified a priori, namely maternal age and preexisting hypertension, was performed. All significance testing was 2-sided, and results were considered statistically significant at *P* < .05. Data were analyzed using Stata version 18.0 (StataCorp). Data were analyzed from January 1 to June 25, 2024

## Results

**Figure 1.**
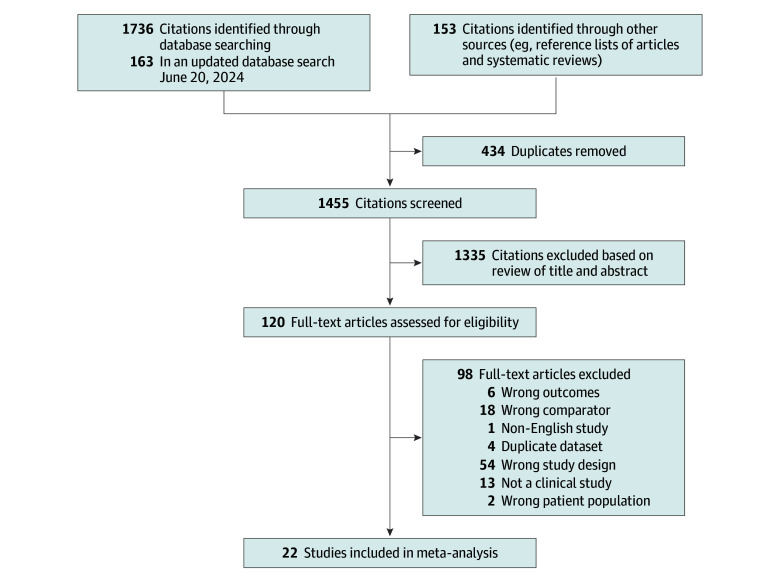
Flow Diagram of Study Selection Process

The search yielded 1736 unique results (Figure 1), and 22 articles^15,16,17,18,19,20,21,22,23,24,25,26,27,28,29,30,31,32,33,34,35,36^ were selected as relevant studies for our analysis, including a total study population of 92 289 079 women (93 565 343 pregnancies). There were a total of 4431 women who had 4786 pregnancies following a solid organ transplant. Of these, 2758 pregnancies (57.6%) were among women with kidney transplants, 1811 pregnancies (37.8%) were among women with liver transplants, 211 pregnancies (4.4%) were among women with heart transplants, and 6 pregnancies (0.1%) were among women with lung transplants.

Of 22 studies, 14 studies^16,17,18,19,20,21,22,23,24,25,26,27,28,36^ (3187 pregnancies in 3090 women with a solid organ transplant) reported at least 1 primary or secondary adverse pregnancy outcome. Thirteen studies^15,16,19,26,27,28,29,30,31,32,33,34,35^ (2000 pregnancies in 1728 women with a solid organ transplant) reported the rate of allograft outcomes during pregnancy.

There were 6 studies from North America,^15,17,20,24,27,36^ 2 studies from Australia,^18,25^ 11 studies from Europe,^16,19,21,22,23,26,29,30,32,33,35^ 1 study from Asia^34^ and 2 studies from the Middle East.^28,31^ The sample size of included studies ranged widely, from 120 women in an Iranian study^28^ to a registry-based study of 38 449 030 US women.^20^

The summary results of all meta-analyses are presented in Table 1. Study characteristics of the 14 articles^16,17,18,19,20,21,22,23,24,25,26,27,28,36^ that provided effect estimates for at least 1 adverse pregnancy outcome are presented in Table 2 (baseline characteristics in eTable 1 in Supplement 1). Study characteristics of the 13 articles^15,16,19,26,27,28,29,30,31,32,33,34,35^ that reported allograft outcomes are presented in eTable 2 in Supplement 1.

**Table 1.** Summary Results of Meta-Analyses

Outcome	Studies, No.	Source	Pregnancies, No.	Outcomes, No.	Pooled OR (95% CI)	*I*^2^, %	τ^2^
**Preeclampsia**							
Crude	11	Barros et al,^16^ 2022; DeFilippis et al,^17^ 2022; Hewawasam et al,^18^ 2022; Mazanowska et al,^19^ 2022; Sobotka et al,^20^ 2021; Madej et al,^22^ 2016; Majak et al,^23^ 2016; Arab et al,^24^ 2015; Bramham et al,^26^ 2013; Coffin et al,^27^ 2010; Pezeshki et al,^28^ 2004	70 069 293	2 861 580	6.34 (4.94 to 8.14)	67.6	0.09
Adjusted (any)	5	DeFilippis et al,^17^ 2022; Hewawasam et al,^18^ 2022; Majak et al,^23^ 2016; Arab et al,^24^ 2015; Bramham et al,^26^ 2013	31 615 223	1 284 691	5.83 (3.45 to 9.87)	77.4	0.25
Adjusted for prespecified covariates^a^	3	DeFilippis et al,^17^ 2022; Hewawasam et al,^18^ 2022; Majak et al,^23^ 2016	24 520 251	1 011 433	4.14 (2.87 to 5.97)	1.7	0.00
**Preterm birth <37 wk**							
Crude	12	Craig et al,^36^ 2023; Barros et al,^16^ 2022; Hewawasam et al,^18^ 2022; Sobotka et al,^20^ 2021; Piccoli et al,^21^ 2017; Madej et al,^22^ 2016; Majak et al,^23^ 2016; Arab et al,^24^ 2015; Wyld et al,^25^ 2013; Bramham et al,^26^ 2013; Coffin et al,^27^ 2010; Pezeshki et al,^28^ 2004	91 105 532	4 919 542	7.48 (4.49 to 12.47)	95.1	0.74
Adjusted (any)	4	Craig et al,^36^ 2023; Majak et al,^23^ 2016; Arab et al,^24^ 2015; Bramham et al,^26^ 2013	46 995 396	3 793 167	6.65 (4.09 to 12.83)	81.8	0.19
Adjusted for prespecified covariates^a^	3	Craig et al,^36^ 2023; Majak et al,^23^ 2016; Arab et al,^24^ 2015	46 933 949	3 793 002	5.31 (3.93 to 7.17)	41.1	0.03
**Low birth weight <2500 g**							
Crude	11	Barros et al,^16^ 2022; DeFilippis et al,^17^ 2022; Hewawasam et al,^18^ 2022; Mazanowska et al,^19^ 2022; Sobotka et al,^20^ 2021; Piccoli et al,^21^ 2017; Majak et al,^23^ 2016; Arab et al,^24^ 2015; Wyld et al,^25^ 2013; Bramham et al,^26^ 2013; Coffin et al,^27^ 2010	73 168 692	1 969 495	5.50 (3.42 to 8.86)	91.7	0.54
Adjusted (any)	4	DeFilippis et al,^17^ 2022; Hewawasam et al,^18^ 2022; Arab et al,^24^ 2015; Bramham et al,^26^ 2013	31 944 922	972 050	6.51 (2.85 to 14.88)	90.6	0.58
Adjusted for prespecified covariates^a^	3	DeFilippis et al,^17^ 2022; Hewawasam et al,^18^ 2022; Arab et al,^24^ 2015	31 943 476	971 894	4.96 (1.60 to 15.37)	92.5	0.82
**Live birth**							
Crude	4	Barros et al,^16^ 2022; Hewawasam et al,^18^ 2022; Wyld et al,^25^ 2013; Bramham et al,^26^ 2013	8 263 840	8 199 030	0.28 (0.03 to 2.61)	97.8	5.02
Adjusted (any)	NA	NA	NA	NA	NA	NA	NA
Adjusted for prespecified covariates^a^	NA	NA	NA	NA	NA	NA	NA
**Preterm birth <32 wk**							
Crude	4	Hewawasam et al,^18^ 2022; Piccoli et al,^21^ 2017; Wyld et al,^25^ 2013; Bramham et al,^26^ 2013	5 717 636	99 507	14.47 (8.29 to 25.28)	80.5	0.25
Adjusted (any)	2	Hewawasam et al,^18^ 2022; Bramham et al,^26^ 2013	2 947 860	55 139	10.35 (6.89 to 15.53)	2.7	0.00
Adjusted for prespecified covariates	1	Hewawasam et al,^18^ 2022	2 946 413	55 103	11.4 (7.41 to 17.55)	NA	NA
**Birth weight <1500 g**							
Crude	5	Hewawasam et al,^18^ 2022; Piccoli et al,^21^ 2017; Madej et al,^22^ 2016; Wyld et al,^25^ 2013; Bramham et al,^26^ 2013	5 717 634	78 258	4.97 (2.28 to 10.82)	87.0	0.67
Adjusted (any)	1	Bramham et al,^26^ 2013	1446	33	7.76 (3.29 to 18.3)	NA	NA
Adjusted for prespecified covariates^a^	0	NA	NA	NA	NA	NA	NA
**Gestational age**							
Crude	5	Barros et al,^16^ 2022; Mazanowska et al,^19^ 2022; Majak et al,^23^ 2016; Wyld et al,^25^ 2013; Pezeshki et al,^28^ 2004	2 769 094	NA	−3.37 (−3.76 to −2.99)^b^	0.0	0.00
Adjusted (any)	0	NA	NA	NA	NA	NA	NA
Adjusted for prespecified covariates^a^	0	NA	NA	NA	NA	NA	NA
**Cesarean delivery**							
Crude	11	Craig et al,^36^ 2023; Barros et al,^16^ 2022; Hewawasam et al,^18^ 2022; Mazanowska et al,^19^ 2022; Sobotka et al,^20^ 2021; Piccoli et al,^21^ 2017; Madej et al,^22^ 2016; Majak et al,^23^ 2016; Arab et al,^24^ 2015; Bramham et al,^26^ 2013; Coffin et al,^27^ 2010	88 293 100	27 376 648	3.67 (2.33 to 5.78)	96.1	0.54
Adjusted (any)	5	Craig et al,^36^ 2023; Hewawasam et al,^18^ 2022; Majak et al,^23^ 2016; Arab et al,^24^ 2015; Bramham et al,^26^ 2013	49 837 531	15 991 969	3.30 (2.13 to 5.11)	85.5	0.21
Adjusted for prespecified covariates^a^	4	Craig et al,^36^ 2023; Hewawasam et al,^18^ 2022; Majak et al,^23^ 2016; Arab et al,^24^ 2015	49 836 076	10 200 179	3.06 (1.89 to 4.94)	86.1	0.20

Abbreviations: NA, not applicable; OR, odds ratio.

^a^ Prespecified comorbidities: maternal age and preexisting hypertension.

^b^ Expressed as mean difference (95% CI) in weeks.

**Table 2.** Characteristics of Studies That Reported at Least 1 Adverse Pregnancy Outcome

Source	Country	Study design, data source	Pregnancies, No.	Exposure	Definition of control group	Outcomes	Confounders adjusted
Craig et al,^36^ 2023	US	Retrospective cohort, National Readmissions Database, HCUP	39 839 192	Heart transplant	Women without a history of heart transplant	Preterm birth, cesarean delivery	Age, comorbid conditions (including hypertension), calendar year, demographics, and facility characteristics
Barros et al,^16^ 2022	Portugal	Retrospective cohort, clinical records at single institution	243	Kidney transplant	Healthy women without transplantation who had antenatal follow up and delivery at the same institution between 2009 and 2019	Preeclampsia, preterm birth <37 wk, LBW <2500 g, gestational age, live birth, cesarean delivery	NA
DeFilippis et al,^17^ 2022	US	Retrospective cohort, National Inpatient Sample, HCUP	21 922 631	Heart transplant	Women without history of heart transplant or systolic heart failure and had hospitalization for delivery	Preeclampsia, preterm birth <37 wk, LBW <2500 g, cesarean delivery	Maternal age, race, preexisting hypertension, kidney failure, diabetes, Elixhauser Comorbidity Index
Hewawasam et al,^18^ 2022	Australia	Retrospective cohort, Australian & New Zealand Dialysis and Transplant Registry and state-level perinatal datasets	2 903 135	Kidney transplant	Babies born to mothers who had either never received KRT or had given birth prior to commencement of KRT	Preeclampsia, LBW <2500 g, preterm birth <32 wk, LBW <1500 g, live birth, cesarean delivery	Maternal age, preexisting hypertension, preexisting diabetes, socioeconomic status, parity, multiple births
Mazanowska et al,^19^ 2022	Poland	Retrospective cohort, clinical records at single institution	137	Kidney transplant	Healthy patients with reference range kidney function matched according to age, BMI, and gestational age	Pregnancy-induced hypertension, gestational age at delivery, LBW, cesarean delivery, intrauterine growth restriction (<10%)	NA
Sobotka et al,^20^ 2021	US	Retrospective cohort, NIS (2005-2013)	38 449 030	LT	Women age >18 y who had hospitalization for delivery and no LT	Preeclampsia, preterm birth <37 wk, LBW <2500 g, cesarean delivery	Maternal age, race, income, type of insurance, Elixhauser Comorbidity Index, and hospital size, type, and region
Piccoli et al,^21^ 2017	Italy	Retrospective cohort, clinical records across multiple sites	731	Kidney transplant	Pregnancies occurring in the absence of hypertension, obesity, diabetes, CKD, cardiovascular disease	Preeclampsia, preterm birth <37 wk, LBW, preterm birth <34 wk, cesarean delivery	NA
Madej et al,^22^ 2016	Poland	Retrospective cohort, clinical records at single institution	288	Kidney transplant or LT	Pregnancies in nontransplanted women	Preeclampsia, preterm birth <37 wk, LBW <2500 g, mean gestational age, cesarean delivery	NA
Majak et al,^23^ 2016	Norway	Retrospective cohort, national registry	357	Kidney transplant	Random sample nontransplanted women who delivered first baby	Preeclampsia, preterm birth, LBW <2500 g, gestational age, cesarean delivery	Hypertension and twin pregnancy
Arab et al,^24^ 2015	US	Retrospective cohort, NIS	7 094 400	Kidney transplant	Deliveries in women without kidney transplant	Preeclampsia, preterm birth <37 wk, cesarean delivery	Maternal age, race, smoking, obesity, preexisting hypertension, diabetes
Wyld et al,^25^ 2013	Australia	Retrospective cohort, Australian & New Zealand Dialysis and Transplant Registry and national perinatal registry	5 270 337	Kidney transplant	All deliveries in Australia 1991-2010	Preeclampsia, preterm birth <37 wk, LBW <2500 g, live birth, mean gestational age	NA
Bramham et al,^26^ 2013	UK	Retrospective cohort, national registry and hospital records at multiple institutions	1465	Kidney transplant	Women without kidney transplant	Preeclampsia, preterm birth <37 wk, LBW <2500 g, live birth, preterm birth <32 wk, LBW <1500 g, cesarean delivery	Maternal age, parity, smoking status
Coffin et al,^27^ 2010	US	Retrospective cohort, national registry (1993-2005)	4266	LT	Randomly selected controls (no LT) matched on age, hospital, and calendar year	Preeclampsia, preterm birth <37 wk, LBW <2500 g, live birth, cesarean delivery	Maternal age, sex, race, insurance, comorbidities, admission characteristics, year, region, LT hospital center
Pezeshki et al,^28^ 2004	Iran	Retrospective cohort, single institution	120	Kidney transplant	Pregnancies in nontransplant patients, matched for age and parity	Preeclampsia, preterm <37 wk, LBW <2500 g, gestational age	NA

Abbreviations: CKD, chronic kidney disease; BMI, body mass index; HCUP, Healthcare Costs and Utilization Project; KRT, kidney replacement therapy; LBW, low birth weight; LT, liver transplant; NA, not applicable; NIS, National Inpatient Sample.

### Preeclampsia

**Figure 2.**
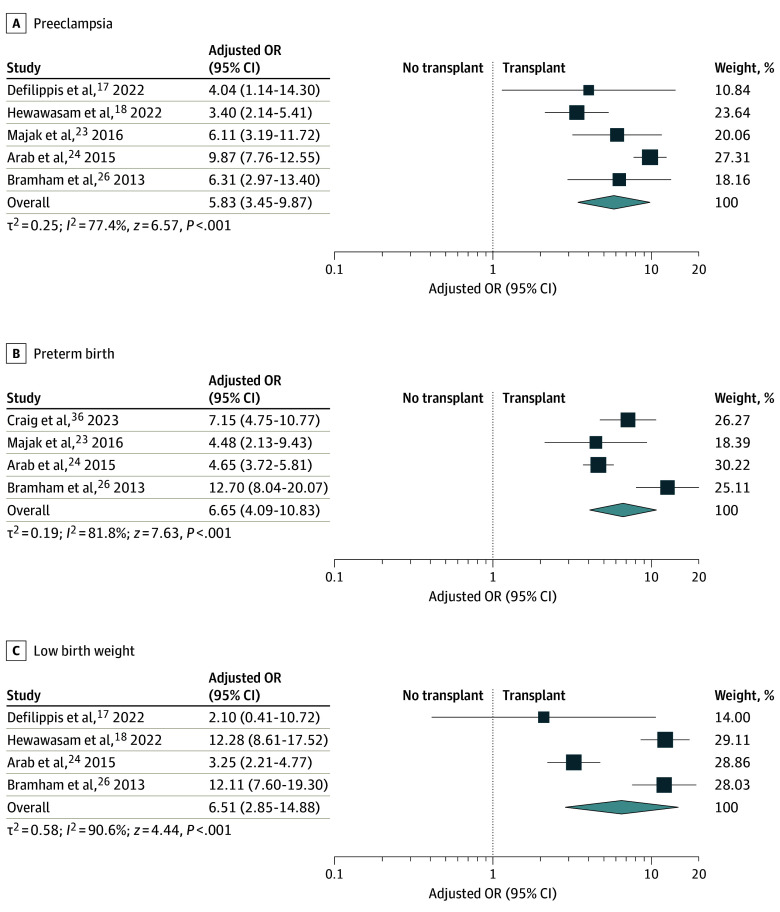
Summary of Pooled Adjusted Odds Ratios (ORs) for the Association Between Solid Organ Transplantation and Adverse Pregnancy Outcomes Preterm birth was defined as birth at less than 37 weeks’ gestation; low birth weight, birth weight less than 2500 g. Size of squares indicates study weight; diamond, overall findings.

Eleven studies^16,17,18,19,20,22,23,24,26,27,28^ provided effect estimates for the association between solid organ transplantation and preeclampsia, with 5 studies^17,18,23,24,26^ also providing adjusted effect estimates. In a meta-analysis, solid organ transplantation was significantly associated with preeclampsia (OR, 6.34 [95% CI, 4.94 to 8.14]; *I*^2^ = 67.6%; aOR, 5.83 [95% CI, 3.45 to 9.87]; *I*^2^ = 77.4%) (Figure 2). In a sensitivity analysis, results were similar when restricting the meta-analysis to the 3 studies^17,18,23^ that adjusted for maternal age and preexisting hypertension (aOR, 4.14 [95% CI, 2.87 to 5.97]; *I*^2^ = 1.7%).

### Preterm Birth 

Twelve studies^16,18,19,20,22,23,24,25,26,27,28,36^ reported estimates of the association between solid organ transplantation and preterm birth and 4 studies^23,24,26,36^ reported adjusted effect estimates. Solid organ transplantation was found to be significantly associated with preterm birth in meta-analysis (OR, 7.48 [95% CI, 4.49 to 12.47]; *I*^2^ = 95.1%; aOR, 6.65 [95% CI, 4.09 to 12.83]; *I*^2^ = 81.8%) (Figure 2). When we limited the meta-analysis to 3 studies^23,24,36^ in a sensitivity analysis, the association between solid organ transplantation and preterm birth was similar (aOR, 5.31 [95% CI, 3.93 to 7.17]; *I*^2^ = 41.1%).

### Low Birth Weight 

Eleven studies^16,17,18,19,20,21,23,24,25,26,27^ reported effect estimates of the association between solid organ transplantation and low birth weight, with 4 studies^17,18,24,26^ also reporting adjusted effect estimates. In a meta-analysis, solid organ transplantation was significantly associated with low birth weight (OR, 5.50 [95% CI, 3.42 to 8.86]; *I*^2^ = 91.7%; aOR, 6.51 [95% CI, 2.85 to 14.88]; *I*^2^ = 90.6%) (Figure 2). Sensitivity analysis was performed with three studies^17,18,24^ that reported effect estimates adjusting for preexisting hypertension and maternal age with similar effects (aOR, 4.96 [95% CI, 1.60 to 15.37]; *I*^2^ = 92.5%).

### Secondary Adverse Pregnancy Outcomes

Four studies^16,18,25,26^ reported effect estimates of the association between solid organ transplantation and live birth. When the effect estimates were pooled, there was no significant association between solid organ transplantation and live birth (OR, 0.28 [95% CI, 0.03 to 2.61]; *I*^2^ = 97.8%) (eFigure 1 in Supplement 1).

Four studies^18,21,25,26^ provided effect estimates of the associations between solid organ transplantation and very preterm birth. Two studies^18,26^ also reported adjusted effect estimates. In meta-analysis, solid organ transplantation was significantly associated with very preterm birth (OR, 14.47 [95% CI, 8.29 to 25.28]; *I*^2^ = 80.5%; aOR, 10.35 [95% CI, 6.89 to 15.53]; *I*^2^ = 2.7%) (eFigure 2 in Supplement 1). Five studies^18,21,22,25,26^ reported effect estimates of the association between solid organ transplantation and very low birth weight (OR, 4.97 [95% CI, 2.28 to 10.82]; *I*^2^ = 87.0%) (eFigure 3 in Supplement 1).

Eleven studies^16,18,19,20,21,22,23,24,26,27,36^ reported effect estimates of the association between solid organ transplantation and cesarean delivery; 5 studies^18,23,24,26,36^ provided adjusted effect estimates. In meta-analysis, there was a significant association between solid organ transplantation and cesarean delivery (OR, 3.67 [95% CI, 2.33 to 5.78]; *I*^2^ = 96.1%; aOR 3.30 [95% CI, 2.13 to 5.11]; *I*^2^ = 85.5%) (eFigure 4 in Supplement 1).

Five studies^16,19,23,25,28^ reported gestation at birth, measured in weeks. In meta-analysis, there was a statistically significant MD in gestational age at birth in pregnancies comparing solid organ transplantation vs no transplantation (MD, −3.37 [95% CI, −3.37 to −2.99] weeks; *I*^2^ = 0.0%) (eFigure 5 in Supplement 1).

Sensitivity analysis for the secondary adverse pregnancy outcomes was only able to be performed for the association between solid organ transplantation and cesarean birth. Four studies^18,23,24,36^ adjusted specifically for preexisting hypertension and maternal age, resulting in a similar effect estimate (aOR, 3.06 [95% CI, 1.89 to 4.94]; *I*^2^ = 86.1%).

### Allograft Outcomes

In 13 studies^15,16,19,26,27,28,29,30,31,32,33,34,35^ (2000 pregnancies in 1728 women with a solid organ transplant) that reported allograft outcomes during pregnancy, most were from kidney transplants (1696 pregnancies [84.8%]), followed by liver transplants (286 pregnancies [14.3%]), lung transplants (6 pregnancies [0.3%]), and heart transplants (12 pregnancies [0.6%]). The pooled incidence of acute allograft rejection during pregnancy was 2.39% (95% CI, 1.20% to 3.96%; I^2^ = 68.5%) and of allograft loss during pregnancy was 1.55% (95% CI, 0.05% to 4.44%; *I*^2^ = 69.2%) (eTable 3, eFigure 6, and eFigure 7 in Supplement 1). Of the 51 episodes of acute rejection, 33 (64.7%) occurred in women with kidney transplants and 18 (35.3%) occurred in women with liver transplants. Nine episodes of allograft loss during pregnancy occurred in women with kidney transplants, with none reported in other organ transplant types.

### Publication Bias and Risk of Bias Assessment

An assessment of publication bias for acute allograft rejection during pregnancy indicated there is likely small study effects (eFigure 8 in Supplement 1). There were too few studies to evaluate small study effects for other outcomes. One study was assessed as low quality^22^ with the remaining studies of high or moderate methodological quality (eTable 4 and eTable 5 in Supplement 1).

## Discussion

In this systematic review and meta-analysis of 22 studies and 93 565 343 pregnancies, solid organ transplantation was significantly associated with adverse pregnancy outcomes. The risks of preeclampsia, preterm birth (<37 weeks), and low birth weight (<2500 g) in pregnant women with a solid organ transplant were 4 to 6 times those of pregnant women without an organ transplant.

To date, this is one of the largest contemporary systematic reviews and meta-analyses to quantify the association between solid organ transplantation and adverse pregnancy outcomes. Results of the meta-analysis support and extend the findings of prior reviews.^4,5,6,37,38^ Shah et al^4^ described high rates of maternal and fetal outcomes in kidney transplant recipients, with almost one-quarter developing preeclampsia and 41% delivering preterm. Previous meta-analyses of pregnancy outcomes in liver and thoracic transplant recipients also reported high pooled incidences of adverse pregnancy outcomes.^6,37,38^

Results of the meta-analysis also show that while there was a high risk of preterm birth, most women delivered in the early term period. The MD in gestational age in a pregnancy from a transplant recipient vs nontransplant control was approximately 3 weeks. This is important new knowledge that will inform clinical practice and also provide reassurance to expectant mothers and health care practitioners.

We did not observe a significant difference in live births; however, there is likely a high level of imprecision in our estimate. Previous meta-analyses have reported higher live birth rates in kidney and liver transplant recipients compared with the live birth rate in the US general population.^4,5,6^ There are a number of potential reasons for this discrepancy. First, the definition of live birth rate is variable and range from a baby born with any signs of life through to any live birth greater than 20 weeks.^16,18^ Second, there is likely underreporting of early pregnancy loss in transplant registries. Third, the rates of termination in this high risk cohort are unknown, particularly where fetal morbidity may potentially have been increased due to exposure to teratogenic medications or maternal comorbidities.

Graft rejection during pregnancy is a major concern due to the limited treatment options available as a result of potential toxic effects for the fetus. The incidence of rejection during pregnancy reported in previous studies ranged between 4.2% to 9.4%, higher than in the our study.^4,5^ These differences may be attributed to the overrepresentation of pregnancies from different transplant eras and the resultant changes in obstetric and transplant practices. Advances in immunosuppressive therapy have improved rejection rates over the past 3 decades.^39^ In addition, the pharmacokinetics of calcineurin inhibitors, a mainstay of immunosuppression regimens, are altered during pregnancy due to changes in drug distribution and metabolism.^40,41^ Greater recognition of the need to uptitrate and maintain therapeutic levels of calcineurin inhibitors during pregnancy may have also contributed to the lower incidence of rejection found in our study.

Several potential mechanisms underlie the association between adverse pregnancy outcomes and solid organ transplantation. First, risk factors, such as obesity, preexisting hypertension, and diabetes, are more common in transplant recipients, which contribute to the increased risk of preeclampsia and fetal growth restriction.^26^ With regards to preterm birth, we do not have pregnancy-specific data to differentiate whether this was spontaneous or medically indicated; however, prior studies have shown that it is primarily due to worsening kidney function, preeclampsia, or severe hypertension.^26,35^ Second, while it is clear that preeclampsia is an important determinant of perinatal outcomes, other unmeasured factors, such as fetal assessment and antenatal kidney function, may further contribute to the high perinatal morbidity seen in this population.^18^ Finally, immunosuppressive medications may play a role in driving the risk of adverse pregnancy outcomes. Calcineurin inhibitors are a known cause of hypertension and endothelial dysfunction.^42,43^ A Dutch study found higher rates of preeclampsia in kidney transplant recipients with calcineurin inhibitors compared with recipients not using calcineurin (40% vs 30%), although this study was limited by small sample size.^44^

Strengths of this systematic review and meta-analysis include the comprehensive literature search, inclusion of studies from contemporary cohorts, and use of adjusted effect estimates and assessment of individual study quality. In addition, sensitivity analysis was performed using a core set of confounders determined a priori to ensure the robustness of our findings.

### Limitations

This study has several limitations. First, only English-language articles were included. Second, high levels of heterogeneity were observed, warranting a cautious interpretation of the results. We were unable to explore reasons for heterogeneity in planned subgroup analysis owing to the small number of studies. Potential reasons for heterogeneity may include maternal factors (eg, interval between transplantation and pregnancy) as well as solid organ transplant type. Third, publication bias remains a possibility, as we were unable to assess for this with respect to the adverse pregnancy outcomes due to the small number of studies. Fourth, the inherent biases of observational studies that constitute the evidence base for this meta-analysis limit interpretations. Although adjustment for confounders was sought, studies varied in how they performed this. Furthermore, there is far more experience with pregnancy in kidney and liver transplants compared with thoracic transplants. Heart and lung transplant recipients have unique maternal risks during pregnancy, including response to the physiologic adaptions associated with pregnancy, higher risk of rejection requiring increased levels of immunosuppression, and comorbidity profiles.^45,46^ These limitations underscore the need for an individualized approach to preconception counselling and risk assessment by an expert multidisciplinary team.

Further research is needed to examine data on the immunosuppression used at time of conception and during pregnancy, as well as effective risk reduction interventions, such as aspirin supplementation for preeclampsia prevention. The minimal data found by our study on early pregnancy losses and reasons for preterm birth during pregnancy support the need for capturing such data in transplant registries to assist with future studies.

## Conclusions

In this systematic review and meta-analysis, pregnancies in solid organ transplant recipients, compared with pregnancies in individuals without an organ transplant, were significantly associated with adverse pregnancy outcomes. The overall rate of allograft rejection and loss during pregnancy was low.

## Notes

Supplement 1. **eAppendix 1.** Search Strategy **eTable 1.** Summary of Baseline Characteristics for Studies Reporting at Least 1 Adverse Pregnancy Outcome **eTable 2.** Study Characteristics of Studies Reporting Allograft Outcomes During Pregnancy **eFigure 1.** Forest Plot for Live Birth **eFigure 2.** Forest Plot for Preterm Birth <32 Weeks (Adjusted Estimate) **eFigure 3.** Forest Plot for Low Birth Weight <1500 g (Crude Estimate) **eFigure 4.** Forest Plot for Caesarean Section (Adjusted Estimate) **eFigure 5.** Forest Plot for Mean Difference in Gestational Age (Weeks) **eTable 3.** Meta-Analysis of Allograft Outcomes During Pregnancy **eFigure 6.** Forest Plot for Allograft Rejection During Pregnancy **eFigure 7.** Forest Plot for Allograft Loss During Pregnancy **eFigure 8.** Funnel Plot for Studies Reporting Allograft Rejection During Pregnancy **eTable 4.** Risk of Bias Assessment for Studies Reporting at Least 1 Adverse Pregnancy Outcome **eTable 5.** Risk of Bias Assessment for Allograft Outcomes

Supplement 2.
Data Sharing Statement
