# Supplementary material for: Adverse Pregnancy Outcomes in Solid Organ Transplant Recipients: A Systematic Review and Meta-Analysis
Source: JAMA Netw Open. 2024 Aug 29;7(8):e2430913. doi: 10.1001/jamanetworkopen.2024.30913 (PMC11362861; doi:10.1001/jamanetworkopen.2024.30913)
Supplement: Supplement 1. — eAppendix 1. Search Strategy eTable 1. Summary of Baseline Characteristics for Studies Reporting at Least 1 Adverse Pregnancy Outcome eTable 2. Study Characteristics of Studies Reporting Allograft Outcomes During Pregnancy eFigure 1. Forest Plot for Live Birth eFigure 2. Forest Plot for Preterm Birth <32 Weeks (Adjusted Estimate) eFigure 3. Forest Plot for Low Birth Weight <1500 g (Crude Estimate) eFigure 4. Forest Plot for Caesarean Section (Adjusted Estimate) eFigure 5. Forest Plot for Mean Difference in Gestational Age (Weeks) eTable 3. Meta-Analysis of Allograft Outcomes During Pregnancy eFigure 6. Forest Plot for Allograft Rejection During Pregnancy eFigure 7. Forest Plot for Allograft Loss During Pregnancy eFigure 8. Funnel Plot for Studies Reporting Allograft Rejection During Pregnancy eTable 4. Risk of Bias Assessment for Studies Reporting at Least 1 Adverse Pregnancy Outcome eTable 5. Risk of Bias Assessment for Allograft Outcomes [file jamanetwopen-e2430913-s001.pdf]

Supplemental Online Content

Yo JH, Fields N, Li W, et al. Adverse pregnancy outcomes in solid organ transplant recipients: a systematic review and meta-analysis. *JAMA Netw Open*. 2024;7(8):e2430913. doi:10.1001/jamanetworkopen.2024.30913

eAppendix 1. Search Strategy
eTable 1. Summary of Baseline Characteristics for Studies Reporting at Least 1 Adverse Pregnancy Outcome
eTable 2. Study Characteristics of Studies Reporting Allograft Outcomes During Pregnancy
eFigure 1. Forest Plot for Live Birth
eFigure 2. Forest Plot for Preterm Birth <32 Weeks (Adjusted Estimate)
eFigure 3. Forest Plot for Low Birth Weight <1500 g (Crude Estimate)
eFigure 4. Forest Plot for Caesarean Section (Adjusted Estimate)
eFigure 5. Forest Plot for Mean Difference in Gestational Age (Weeks)
eTable 3. Meta-Analysis of Allograft Outcomes During Pregnancy
eFigure 6. Forest Plot for Allograft Rejection During Pregnancy
eFigure 7. Forest Plot for Allograft Loss During Pregnancy
eFigure 8. Funnel Plot for Studies Reporting Allograft Rejection During Pregnancy
eTable 4. Risk of Bias Assessment for Studies Reporting at Least 1 Adverse Pregnancy Outcome
eTable 5. Risk of Bias Assessment for Allograft Outcomes

This supplemental material has been provided by the authors to give readers additional information about their work.

eAppendix 1. Search Strategy

A systematic literature search for studies was conducted on 2 August 2021 in MEDLINE and EMBASE on Ovid and in the Scopus database. All searches were re-run on 14 November 2023 to update the result set. The initial search strategy was tested and optimised in Ovid MEDLINE before translation to additional databases. The results were limited to suitable study designs and articles in English from 2000 onwards. In addition to searching citation databases, reference checking and citation searching was conducted on six studies selected for their relevance. Two authors manually searched five key journals across transplant medicine and nephrology.

The hand searched journals were American Journal of Transplantation, Clinical journal of American society of nephrology, Transplantation, Clinical transplantation and Kidney international as well as selected citations.

EMBASE

1. transplantation/ or graft recipient/ or Organ transplantation/ or Heart transplantation/ or Kidney transplantation/ or Liver transplantation/ or Lung transplantation/ or Pancreas transplantation/
2. solid organ transplant*.tw.
3. (solid organ adj3 (transplant* or allograft* or graft* or allotransplantation)).tw.
4. (transplant* adj (organ or heart* or cardiac or kidney* or renal or liver* or lung or pancre*)).tw.
5. ((allograft* or graft* or allotransplantation) and (organ or heart* or cardiac or kidney* or renal or liver* or lung or pancre*)).mp.
6. 1 or 2 or 3 or 4 or 5
7. exp pregnancy complication/ or Pregnancy outcome/ or Birth Weight/ or high risk pregnancy/ or Stillbirth/ or spontaneous abortion/ or imminent abortion/ or preeclampsia/ or premature labor/
8. (pregnan* adj3 (adverse or complication* or high risk or outcome*)).tw.
9. ((pregnan* or obstetric* or maternal) adj5 (toxemia* or hyper?tension or gestosis or edema or pre?eclampsia or pre?eclamtic or eclamsia or pet or uterine complication* or placental abruption or diabetes or mellitus)).tw.
10. ((birth or child?birth or delivery or labour or labor or parturition) adj5 (adverse or complication* or preterm or prematur* or prem or c?section or caesarean)).tw.
11. ((infant or newborn or neonat* or perinatal or post?neonatal or postpartum) adj5 (mortalit* or death or birth weight or low weight)).tw.
12. (live birth rate or still?birth or still?born or spontaneous abortion or miscarriage).tw.
13. (gestational complication* or gestational age).tw.
14. (pregnan* and (allograft loss or (reject* adj2 (allograft or graft or transplant*)))).tw.
15. exp Pregnancy/ and (Graft Rejection/ or Graft Survival/)
16. 7 or 8 or 9 or 10 or 11 or 12 or 13 or 14 or 15
17. 6 and 16
18. limit 17 to (yr="2000 -Current" and english)
19. exp case control study/ or exp cohort analysis/ or exp cross-sectional study/
20. ((cohort or case?control or follow up or observational) adj (studies or study or analy*)).mp.
21. 19 or 20
22. 18 and 21

Medline

1. Transplants/ or Transplant Recipients/ or Organ transplantation/ or Heart transplantation/ or Kidney transplantation/ or Liver transplantation/ or Lung transplantation/ or Pancreas transplantation/
2. solid organ transplant*.tw.
3. (solid organ adj3 (transplant* or allograft* or graft* or allotransplantation)).tw.
4. (transplant* adj (organ or heart* or cardiac or kidney* or renal or liver* or lung or pancre*)).tw.
5. ((allograft* or graft* or allotransplantation) and (organ or heart* or cardiac or kidney* or renal or liver* or lung or pancre*)).mp.
6. 1 or 2 or 3 or 4 or 5
7. Pregnancy/ or exp Pregnancy complications/ or Pregnancy outcome/ or Birth Weight/ or Pregnancy, High-Risk/ or Stillbirth/ or Abortion, spontaneous/ or Abortion, threatened/ or Pre-eclampsia/ or exp Obstetric labor, premature/
8. (pregnan* adj3 (adverse or complication* or high risk or outcome*)).tw.
9. ((pregnan* or obstetric* or maternal) adj5 (toxemia* or hyper?tension or gestosis or edema or pre?eclampsia or pre?eclamtic or eclamsia or pet or uterine complication* or placental abruption or diabetes or mellitus)).tw.
10. ((birth or child?birth or delivery or labour or labor or parturition) adj5 (adverse or complication* or preterm or prematur* or prem or c?section or caesarean)).tw.
11. ((infant or newborn or neonat* or perinatal or post?neonatal or postpartum) adj5 (mortalit* or death or birth weight or low weight)).tw.
12. (live birth rate or still?birth or still?born or spontaneous abortion or miscarriage).tw.
13. (gestational complication* or gestational age).tw.
14. (pregnan* and (allograft loss or (reject* adj2 (allograft or graft or transplant*)))).tw.
15. exp Pregnancy/ and (Graft Rejection/ or Graft Survival/)
16. 7 or 8 or 9 or 10 or 11 or 12 or 13 or 14 or 15
17. 6 and 16
18. limit 17 to (yr="2000 -Current" and english)
19. exp case-control studies/ or exp cohort studies/ or controlled before-after studies/ or cross-sectional studies/ or historically controlled study/ or Epidemiologic Studies/
20. ((cohort or case?control or follow up or observational) adj (studies or study or analy*)).mp.
21. 19 or 20
22. 18 and 21

eTable 1. Summary of baseline characteristics for studies reporting at least one adverse pregnancy outcome

Reference	With transplant							Without transplant				
	N	Mean age (yr)	Interval transplant pregnancy (yr)	Baseline SCr (μmol/L)	CNI (%)	Preexisting HTN (%)	Preexisting diabetes (%)	N	Mean age (yr)	Baseline SCr (μmol/L)	Preexisting HTN (%)	Preexisting diabetes (%)
Craig et al,[36] 2023	199	27.6	-	-	-	17.9	14.2	39 838 993	28.4	-	2.0	1.1
Barros et al,[16] 2022	43	30.6	4.6	114.1	65.0	58.1	5.4	200	32.5	-	1.0	0.5
DeFilippis et al,[17] 2022	94	26.1	-	-	-	32.0	11.0	21 922 537	28.1	-	0.1	1.0
Hewawasam et al,[18] 2022	202	32.4	6	103	-	22.8	4.5	2 902 933	29.0	-	0.9	0.5
Mazanowska et al,[19] 2022	27	35.0	8.9	91.1	96.3	77.8	-	110	34.1	-	0.0	0.0
Sobotka et al,[20] 2021	1469	27.2	-	-	-	-	-	38 447 561	28.0	-	-	-
Piccoli et al,[21] 2017	121	34.1	5.2	94.6	92.5	55.0	-	1 418	31.2	-	0.0	0.0
Madej et al,[22] 2016	101	KT 29.6, LT 28.6	KT 4.5, LT 6.9	-	46.6	KT 71.0, LT 16.0	-	187	29.6	-	-	-
Majak et al,[23] 2016	119	30.3	-	-	-	31.9	5.0	238	25.9	-	0.0	0
Arab et al,[24] 2015	375	-	-	-	-	32.0	10.1	7 094 025	-	-	1.3	1.0
Wyld et al,[25] 2013	692	31	5.3	106	89.0	-	-	5 269 645	30	-	-	-
Bramham et al,[26] 2013	105	32	5	118	91.6	-	-	1 360	-	-	-	-

Reference	With transplant							Without transplant				
	N	Mean age (yr)	Interval transplant pregnancy (yr)	Baseline SCr (µmol/L)	CNI (%)	Preexisting HTN (%)	Preexisting diabetes (%)	N	Mean age (yr)	Baseline SCr (µmol/L)	Preexisting HTN (%)	Preexisting diabetes (%)
Coffin et al,[27] 2010	206	24	-	-	-	2.9	1.5	4 060	25	-	0.0	0.0
Pezeshki et al,[28] 2004	20	29.2	3.0	-	100	-	-	100	28.0	-	-	-

Abbreviations: N, total number of pregnancies; SCr, Serum Creatinine; CNI, Calcineurin inhibitor; HTN, Hypertension; KT, Kidney transplant; LT, Liver transplant

eTable 2. Study characteristics of studies reporting allograft outcomes during pregnancy

Author, year published	Country	Period	Study design	Pregnancies, n
Yin et al,[15] 2023	United States	2000-2019	Retrospective cohort	638
Barros et al,[16] 2022	Portugal	1991-2019	Retrospective cohort	43
Gosselink et al,[29] 2022	Netherlands	1971-2017	Retrospective cohort	288
Mazanowska et al,[19] 2022	Poland	2009-2020	Retrospective cohort	27
Scharwz et al,[30] 2022	Germany	1972-2019	Retrospective cohort	92
Yuksel et al, [31] 2017	Turkey	2009-2016	Retrospective cohort	25
Stoumpos et al,[32] 2016	United Kingdom	1970-2014	Retrospective cohort	138
Blume et al,[33] 2013	Germany	1988-2010	Retrospective cohort	115
Bramham et al,[26] 2013	United Kingdom	2007-2009	Retrospective cohort	105
Coffin et al,[27] 2010	United States	1993-2005	Retrospective cohort	206
Kim et al,[34] 2008	South Korea	1991-2005	Retrospective cohort	74
Sibanda et al,[35] 2007	United Kingdom	1994-2001	Retrospective cohort	229
Pezeshki et al,[28] 2004	Iran	1991-1998	Retrospective cohort	20

eFigure 1. Pooled crude odds ratio for the association between solid organ transplantation and live birth

Study	OR (95% CI)	Weight (%)
Barros et al,[16] 2022	0.14 (0.04 - 0.41)	24.29
Hewawasam et al,[18] 2022	0.20 (0.09 - 0.42)	25.12
Wyld et al,[25] 2013	3.20 (2.68 - 3.80)	25.82
Bramham et al,[26] 2013	0.06 (0.03 - 0.16)	24.77
**Overall**	0.28 (0.03 - 2.61)	

$\tau^2 = 5.02$, $I^2 = 97.8\%$

$z = -1.12$, $p=0.26$

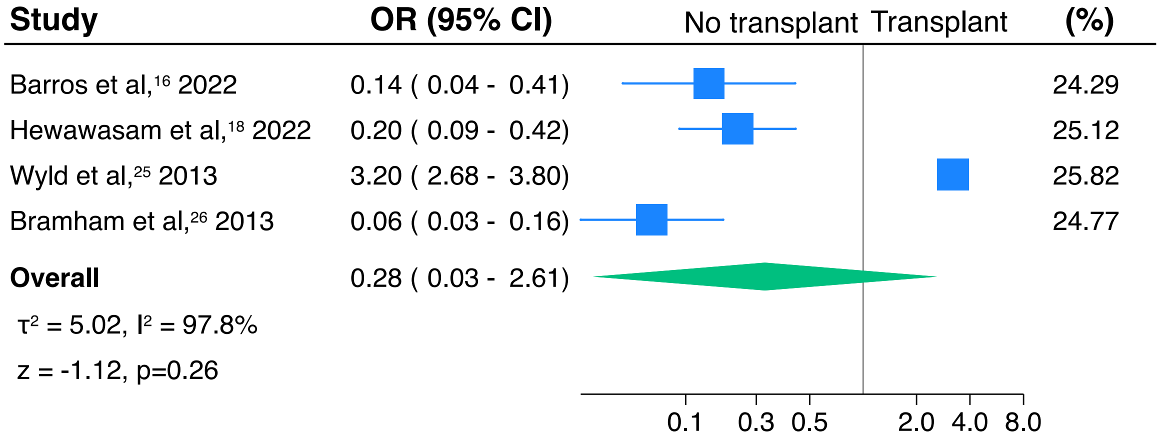

eFigure 2. Pooled adjusted odds ratio for the association between solid organ transplantation and preterm birth < 32 weeks

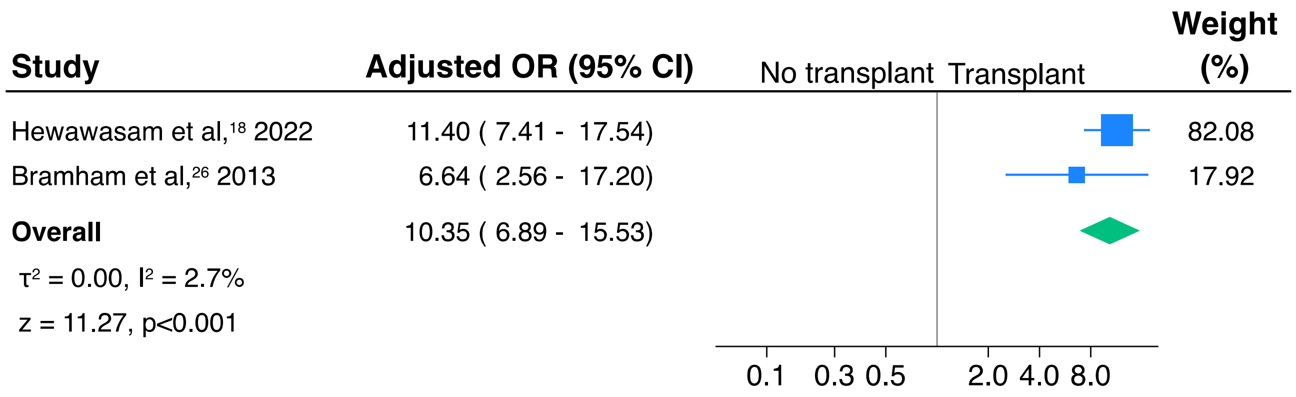

eFigure 3. Pooled crude odds ratio for the association between solid organ transplantation and low birth weight <1500g

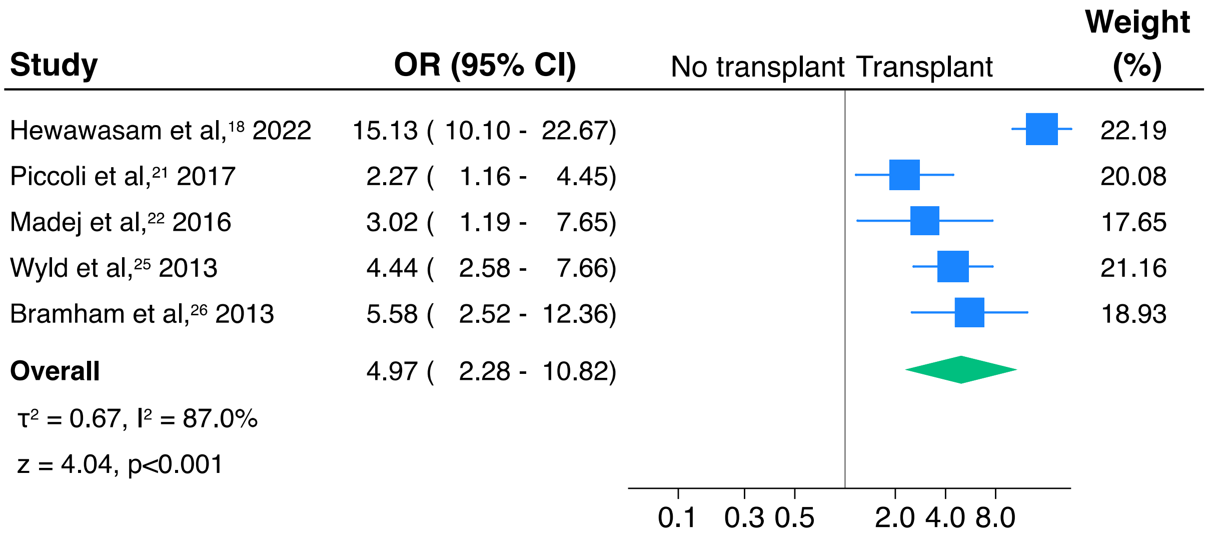

eFigure 4. Pooled adjusted odds ratio for the association between solid organ transplantation and caesarean section

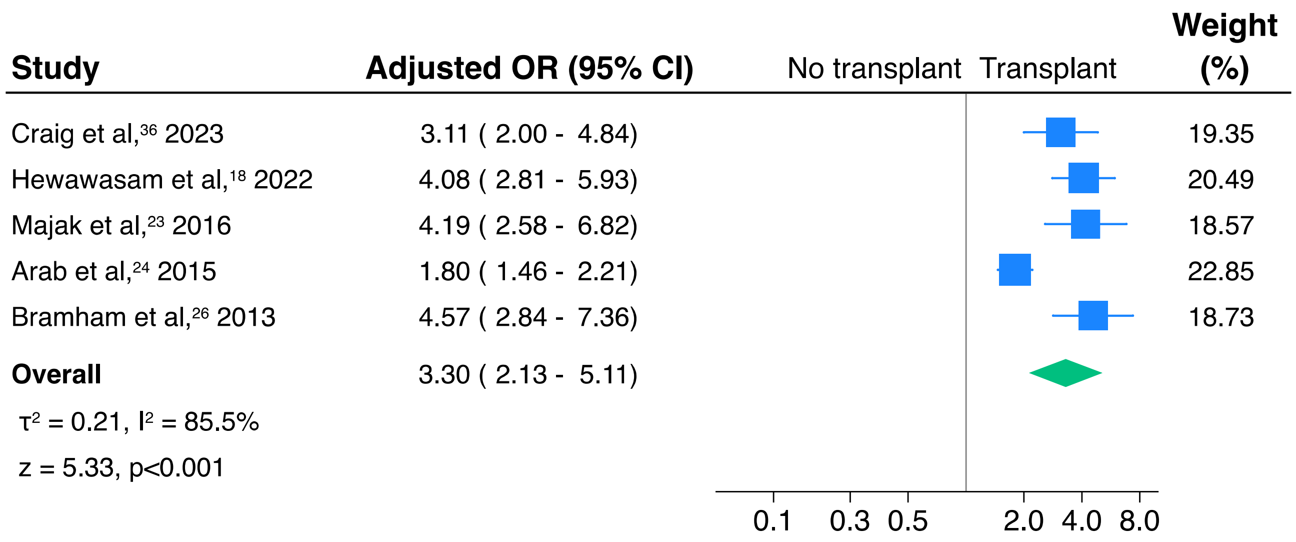

eFigure 5. Pooled mean difference in gestational age

Study	Mean difference (95% CI)	Weight (%)
Barros et al, 2022	-3.40 (-4.29 to -2.51)	18.90
Mazanowska et al, 2022	-4.30 (-5.91 to -2.69)	5.71
Majak et al, 2016	-3.10 (-3.71 to -2.49)	39.34
Wyld et al, 2013	-3.50 (-4.16 to -2.84)	33.96
Pezeshki et al, 2004	-3.60 (-6.27 to -0.93)	2.09
**Overall**	-3.37 (-3.76 to -2.99)	

$\tau^2 = 0.00$, $I^2 = 0.0\%$

$z = -17.2$, $p < 0.001$

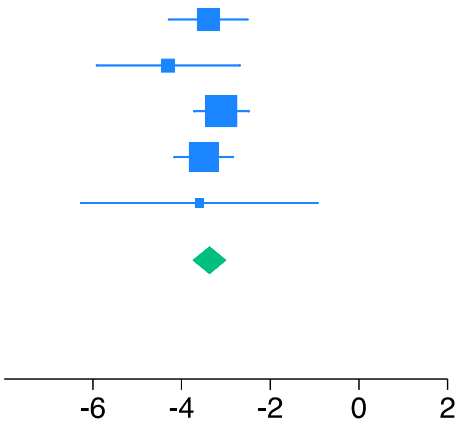

Gestational age was measured in weeks

eTable 3. Meta-analysis of allograft outcomes during pregnancy

Allograft outcomes	No. of studies	Studies	No. of pregnancies	No. of outcomes	Pooled incidence %, (95% CI)	I^2, %	τ^2
Acute rejection during pregnancy	13	Yin et al,[15] 2023<br>Barros et al,[16] 2022<br>Gosselink et al,[29] 2022<br>Mazanowska et al,[19] 2022<br>Scharwz et al,[30] 2022<br>Yuksel et al,[31] 2017<br>Stoumpos et al,[32] 2016<br>Blume et al,[33] 2013<br>Bramham et al,[26] 2013<br>Coffin et al,[27] 2010<br>Kim et al,[34] 2008<br>Sibanda et al,[35] 2007<br>Pezeshki et al,[28] 2004	2000	51	2.39 (1.20-3.96)	68.5	0.020
Acute graft loss during pregnancy	6	Scharwz et al,[30] 2022<br>Yuksel et al,[31] 2017<br>Stoumpos et al,[32] 2016<br>Bramham et al,[26] 2013<br>Sibanda et al,[35] 2007<br>Pezeshki et al,[28] 2004	530	9	1.55 (0.05-4.44)	69.2	0.030

eFigure 6. Pooled acute allograft rejection incidence during pregnancy

Study	Acute rejection	Pregnancies	Acute rejection % (95% CI)	Weight (%)
Yin et al,[15] 2023	14	638	2.19 (1.18 - 3.49)	12.01
Barros et al,[16] 2022	0	43	0.00 (0.00 - 3.96)	5.32
Gosselink et al,[29] 2022	3	288	1.04 (0.13 - 2.62)	10.81
Mazanowska et al,[19] 2022	0	27	0.00 (0.00 - 6.27)	3.95
Schwarz et al, [30] 2022	5	92	5.43 (1.56 - 11.17)	7.79
Yuksel et al,[31] 2017	0	25	0.00 (0.00 - 6.77)	3.74
Stoumpos et al,[32] 2016	6	138	4.35 (1.47 - 8.50)	9.02
Blume et al,[33] 2013	10	115	8.70 (4.14 - 14.63)	8.48
Bramham et al,[26] 2013	2	105	1.90 (0.03 - 5.65)	8.20
Coffin et al,[27] 2010	10	206	4.85 (2.28 - 8.27)	10.08
Kim et al,[34] 2008	1	74	1.35 (1.04 - 5.71)	7.08
Sibanda et al,[35] 2007	0	229	0.00 (0.00 - 0.75)	10.32
Pezeshki et al,[28] 2004	0	20	0.00 (0.01 - 8.43)	3.19
**Overall**			2.39 (1.20 - 3.96)	

$\tau^2 = 0.02$, $I^2 = 68.46\%$

$z = 4.18$, $p<0.0001$

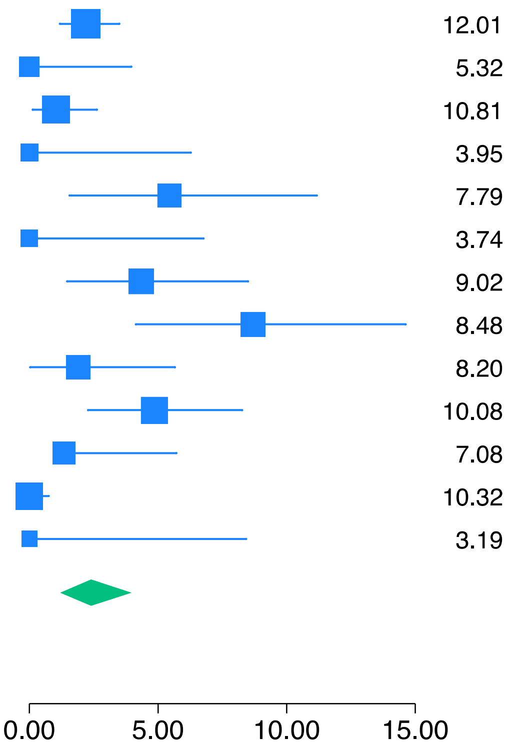

eFigure 7. Pooled acute allograft loss incidence during pregnancy

Study	Acute graft loss	Pregnancies	Acute graft loss % (95% CI)	Weight (%)
Yin et al,[15] 2023	14	638	2.19 (1.18 - 3.49)	12.01
Barros et al,[16] 2022	0	43	0.00 (0.00 - 3.96)	5.32
Gosselink et al,[29] 2022	3	288	1.04 (0.13 - 2.62)	10.81
Mazanowska et al,[19] 2022	0	27	0.00 (0.00 - 6.27)	3.95
Schwarz et al,[30] 2022	5	92	5.43 (1.56 - 11.17)	7.79
Yuksel et al,[31] 2017	0	25	0.00 (0.00 - 6.77)	3.74
Stoumpos et al,[32] 2016	6	138	4.35 (1.47 - 8.50)	9.02
Blume et al,[33] 2013	10	115	8.70 (4.14 - 14.63)	8.48
Bramham et al,[26] 2013	2	105	1.90 (0.03 - 5.65)	8.20
Coffin et al,[27] 2010	10	206	4.85 (2.28 - 8.27)	10.08
Kim et al,[34] 2008	1	74	1.35 (1.04 - 5.71)	7.08
Sibanda et al,[35] 2007	0	229	0.00 (0.00 - 0.75)	10.32
Pezeshki et al,[28] 2004	0	20	0.00 (0.01 - 8.43)	3.19
**Overall**			2.39 (1.20 - 3.96)	

$\tau^2 = 0.03$, $I^2 = 69.2\%$

$z = 2.26$, $p=0.02$

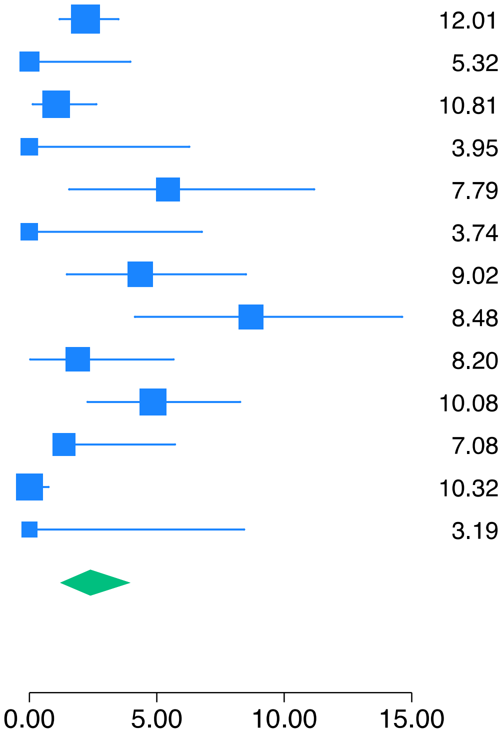

eFigure 8. Funnel plot for studies reporting acute allograft rejection during pregnancy

Eggers test p value = 0.009 indicates small study effects

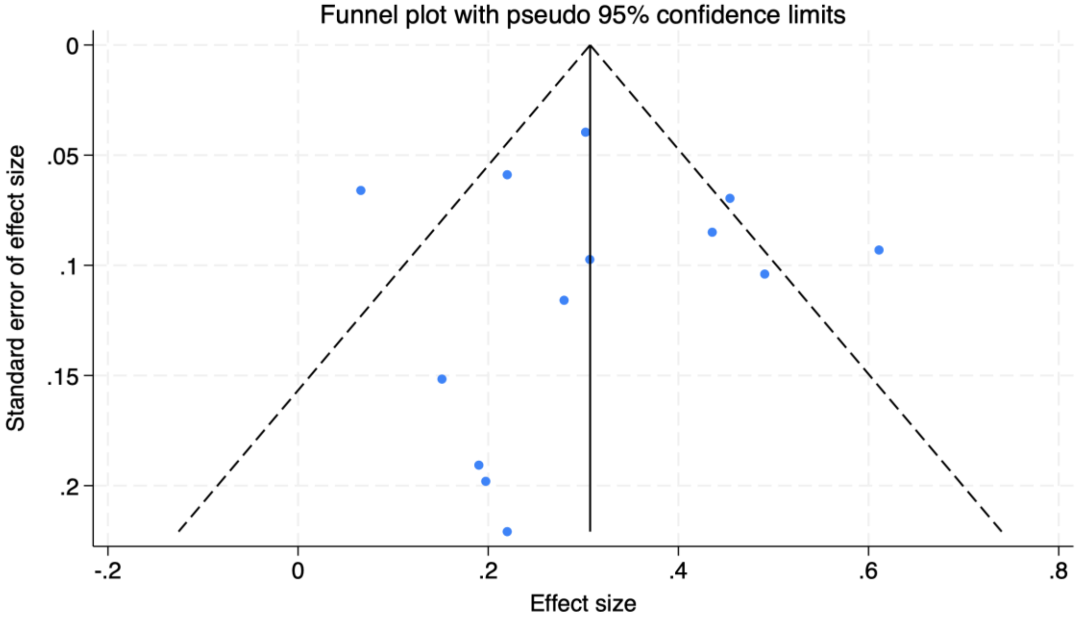

eTable 4. Risk of bias assessment for studies reporting at least one adverse pregnancy outcome

Primary author, Year	Type of study	Representativeness of the exposed cohort	Selection of the non-exposed cohort	Ascertainment of exposure	Demonstration that outcome of interest was not present at the start of the study	Comparability	Ascertainment of outcome	Follow up long enough	Adequacy of follow up	Total Score
Craig et al,[36] 2023	RC	★	★	★	★	★★	★	★	★	9
Barros et al,[16] 2022	RC	★	★	★	★		★	★	★	7
DeFilippis et al,[17] 2022	RC	★	★	★	★	★★	★	★	★	9
Hewawasam et al,[18] 2022	RC	★	★	★	★	★★	★	★	★	9
Mazanowska et al,[19] 2022	RC	★	★	★	★	★	★	★	★	8
Sobotka et al,[20] 2021	RC	★	★	★	★	★	★	★	★	8
Piccoli et al,[21] 2017	RC	★	★	★	★	★★	★	★	★	9
Madej et al,[22] 2016	RC	★		★	★		★	★		5
Majak et al,[23] 2016	RC	★	★	★	★	★★	★	★	★	9
Arab et al,[24] 2015	RC	★	★	★	★	★★	★	★	★	9
Wyld et al,[25] 2013	RC	★	★	★	★	★★	★	★	★	9
Bramham et al,[26] 2013	RC	★	★	★	★	★	★	★	★	8
Coffin et al, [27] 2010	RC	★	★	★	★	★	★	★	★	8
Pezeshki et al, [28] 2004	RC	★		★	★	★	★	★	★	7

Based on Newcastle Ottawa Scale (NOS), the studies were classified as high (8–9 stars), moderate (6–7 stars), and low quality (<6 stars); RC, retrospective cohort study;

eTable 5. Risk of Bias assessment for allograft outcomes

Primary author, Year	Type of study	Representative-ness of the exposed cohort	Selection of the non-exposed cohort	Ascertainment of exposure	Demonstration that outcome of interest was not present at the start of the study	Ascertainment of outcome	Follow up long enough	Adequacy of follow up	Total Score
Yin et al,[15] 2023	RC	★	★	★	★	★	★	★	7
Barros et al,[16] 2022	RC	★	★	★	★	★	★	★	7
Gosselink et al,[29] 2022	RC	★	★	★	★	★	★	★	7
Mazanowska et al,[19] 2022	RC	★	★	★	★	★	★	★	7
Scharwz et al,[30] 2022	RC	★	★	★	★	★	★	★	7
Yuksel et al, [31] 2017	RC	★	★	★	★	★	★	★	7
Stoumpos et al,[32] 2016	RC	★	★	★	★	★	★	★	7
Blume et al,[33] 2013	RC	★	★	★	★	★	★	★	7
Bramham et al,[26] 2013	RC	★	★	★	★	★	★	★	7
Coffin et al,[27] 2010	RC	★	★	★	★	★	★	★	7
Kim et al,[34] 2008	RC	★	★	★	★		★	★	6
Sibanda et al,[35] 2007	CC	★	★	★	★	★	★	★	7
Pezeshki et al,[28] 2004	RC	★		★	★	★	★	★	6

RC, Retrospective cohort study; CC, Case control study
The individual components of the selection (3) and outcome (3) criteria listed above are summed to generate a total modified Newcastle-Ottawa Scale (NOS) risk of bias score for each study. Total scores range from 0 to 6. Studies were judged to be of low risk of bias (≥4 stars) and high risk of bias (<4 stars)
