# Supplementary material for: Adverse Pregnancy Outcomes in Solid Organ Transplant Recipients: A Systematic Review and Meta-Analysis
Source: JAMA Netw Open. 2024 Aug 29;7(8):e2430913. doi: 10.1001/jamanetworkopen.2024.30913 (PMC11362861; doi:10.1001/jamanetworkopen.2024.30913)
Supplement: Supplement 2. — Data Sharing Statement [file jamanetwopen-e2430913-s002.pdf]

Data Sharing Statement

Yo. Adverse Pregnancy Outcomes in Solid Organ Transplant Recipients. *JAMA Netw Open*. Published August 29, 2024. doi:10.1001/jamanetworkopen.2024.30913

Data
Data available: Yes
Data types: Other (please specify)
Additional Information: Statistical code
How to access data: Statistical code will be available upon request
When available: With publication

Supporting Documents
Document types: Other (please specify)
Additional Information: statistical code
How to access documents: upon request of first author - Jennifer Yo
When available: With publication

Additional Information
Who can access the data: List of excluded studies from systematic review
Types of analyses: for purposes of research
Mechanisms of data availability: with investigator support
